# A Strange Use of Hypnotism

**Published:** 1889-11

**Authors:** 


### A Strange Use of Hypnotism.

Nothing more singular could be imagined than the observations related soberly by some of the French experimenters in hypnotism. The remarkable assertions in regard to the application of hypnotism to therapeutic ends are quite matched by those in regard to its manifestations when carried out experimentally, and with a view to determining what actions can be prompted in the hypnotic state. In various places attention has been called to the possibility that criminal actions may be unconsciously performed by persons to whom they have been suggested while hypnotized, and recent reports from Paris indicate that hypnotic subjects may be prompted to indecency and beastiality in the same manner.

For example, in the *Bulletin Medical*, September 4, 1889, it is stated that Tessie claims that he has been able to fix a stamp upon the mind which results in the formation of what he calls "ideogenous zones," or regions, the stimulation of any one of which will be followed by actions of a definite character. His experiments were made in connection with a propensity which is perhaps exceptionally developed in his own country—namely, to indulge the sexual appetite. He describes a case in which he put a man into the hypnotic state, and said to him: "The right auricle represents lubricity, and the left auricle represents chastity." He then waked the subject; whereupon, keeping the most absolute silence, he pressed the patient's right auricle, and saw that he became gradually erotic. At the moment when he was about to masturbate, Tessie pressed the left auricle, and the subject gradually lost his passion and resumed the air of chastity.

The first time Tessie performed this experiment, he says, he forgot to suppress the suggestion. The next day the patient presented himself fatigued, and said that in the evening he had met a friend who had pressed his right hand. Some moments later he became possessed with a violent desire for coitius. He felt that this desire came to him from the right auricle which he pressed several times in the twenty-four hours. He relieved himself by masturbating several times, and that night he had intercourse several times with his mistress; and that very day, on coming to Tessie, he had had two ejaculations, for in walking he had pressed the right auricle.

A story of this sort would hardly be credited were it not told on good authority. It is almost incredible that a scientific investigator should choose such a field for experiment, and one who reflects on the story can not fail to note the character of the subject and to suspect that he merely seized a ready excuse for the indulgence of a propensity to vice, or deliberately played upon the credulity of his investigator.

If there is any element of scientific truth in the singular claim of Tessie that he can "create" ideogenous zones, it is to be hoped that he, and those who imitate him, may hereafter create virtuous and manly zones, and not start their subjects in courses which it may not be so easy to stop.

